# Role of necroptosis-related genes in immune activity and prognosis of colorectal cancer

**DOI:** 10.3389/fimmu.2025.1619749

**Published:** 2025-09-01

**Authors:** Lulu Tan, Shuaifeng Wang, Weilong Chang, Xiaoying Zhang, Rui Deng, Huifang Yan, Weiwei Zhu, Huifen Wang, Yudie Cai, Zhibo Liu, Yuyan Tan, Jinyuan Cui

**Affiliations:** ^1^ Department of Gastrointestinal Surgery, The First Affiliated Hospital of Zhengzhou University, Zhengzhou, China; ^2^ Department of Breast and Thyroid Surgery, The First College of Clinical Medical Science, China Three Gorges University, Yichang, China; ^3^ Gene Hospital of Henan Province, First Affiliated Hospital of Zhengzhou University, Zhengzhou, China; ^4^ The First College of Clinical Medical Science, China Three Gorges University, Yichang, China; ^5^ Department of Radiotherapy, The Second Affiliated Hospital of Zhengzhou University, Zhengzhou, China; ^6^ Department of Infectious Diseases, The First Affiliated Hospital of Zhengzhou University, Zhengzhou, China

**Keywords:** necroptosis, colorectal cancer, prognosis, drug resistance, gene signature, tumor microenvironment

## Abstract

**Background:**

Necroptosis plays a critical role in the onset and progression of numerous malignancies, with colorectal cancer (CRC) ranking among the leading causes of cancer-related mortality worldwide. However, the relationship between necroptosis-related genes (NRGs) and CRC remains contentious. Hence, this study aims to develop a novel NRG-based signature to predict the prognosis of CRC patients and explore its potential role.

**Methods:**

Transcriptome data from the Gene Expression Omnibus (GEO) databases and the Cancer Genome Atlas (TCGA) were employed to identify cancer hallmarks associated with outcomes in CRC. A novel NRG signature was formulated and validated using least absolute shrinkage and selection operator (LASSO) regression analysis and COX regression analysis. Subsequently, univariate and multivariate Cox regression analyses, Kaplan-Meier (K-M) survival analysis, receiver operating characteristic (ROC) curves, and nomograms were utilized to assess the predictive capability of our signature. Furthermore, a variety of bioinformatics analysis algorithms were leveraged to uncover potential mechanisms, tumor immune status, and differences in drug sensitivity between the two-risk groups. The expression of signature NRGs in CRC was evaluated through quantitative reverse transcription polymerase chain reaction (qRT-PCR).

**Results:**

A novel signature consisting of eighteen NRGs (CTSB, PAEP, ARL4C, TAP2, WFS1, BATF2, DUSP27, CXCL9, EPHB2, IRF8, CXCL13, GZMB, APOL6, NLRC5, CXCL10, IRF1, HES6, and PTGDR) was successfully established. This signature displayed consistent predictive performance and general applicability for CRC, as validated across three independent cohorts. Moreover, stromal and immune cells within the tumor microenvironment (TME) were found to be correlated with necroptosis. Additionally, notable differences in the sensitivity to anti-tumor agents were observed between the two groups. The qRT-PCR results indicated aberrant expression of these signature NRGs in CRC.

**Conclusion:**

NRG was proved to be an accurate predictor of CRC prognosis. Furthermore, the novel signature exhibited consistent value and translational potential for predicting prognosis, tumor immunogenicity, and therapeutic response in CRC.

## Introduction

Colorectal cancer (CRC) is a complex malignant disease, with the majority of patients being diagnosed at advanced stages (1). Despite rapid developments in treatment strategies for CRC, including immunotherapy and molecular targeted therapy, the outcomes for CRC patients remain unsatisfactory (2–4). The 5-year overall survival (OS) rate for individuals at advanced stages is less than 10% (5). Consequently, it is evident that early classification, established through thorough and precise risk estimation, is critical for effective CRC treatment. While TNM stratification is widely used to assess and monitor the risk condition of CRC primarily based on clinicopathological parameters (6), its predictive confidence in assessing patients’ prognosis is limited due to the high heterogeneity of CRC (6, 7). Therefore, there is an urgent need to integrate clinicopathological features of the genome for individual survival outcome estimation.

Necroptosis, a previously unidentified programmed cell death pathway independent of the caspase family and distinct from apoptosis (8), manifests morphologically as cell swelling and rounding, explosive plasma membrane potential, and cell membrane perforation when caspase-8 expression is inhibited or cells exhibit low apoptosis. Mechanistically, the formation of the RIP1-RIP3 complex, with receptor-interacting protein (RIP) 1 recruiting RIP3, serves as a pseudokinases complex. Subsequently, this complex phosphorylates its substrate, the mixed lineage kinase domain-like protein (MLKL), leading to the formation of necrosomes and necroptosis (9–12). Emerging evidence suggests that necroptosis could play a dual role in the development of cancer progression and immune surveillance (13–15). Crucial modulators of necroptosis have been reported to promote tumorigenesis and progression, while also impeding tumor growth and metastasis (16–18). For instance, RIP kinase 3 (RIPK3) and RIPK1 have been demonstrated to have anti-tumorigenic capacity in CRC (19), although RIPK3-mediated inflammation can induce an immune-suppressive tumor microenvironment (TME), thereby facilitating intestinal tumor progression (19). Additionally, MLKL has been reported to possess a tumor-suppressive effect during intestinal oncogenesis in certain research, yet the genetic deletion of MLKL showed no influence on CRC advancement (20, 21). This discrepancy is thought to arise from the fact that cells undergoing necroptosis secrete various regulatory cytokines, which can either enhance neoplastic progression by boosting the proliferation of adjacent tumor cells or activate the immunological effect of the TME to eliminate cancer cells. Moreover, triggering necroptosis represents a viable immunotherapeutic option for eradicating tumor cells with apoptosis resistance. Numerous clinical medicines, chemotherapeutic agents, and immunotherapeutic agents have entered clinical trials for specific tumors (18). However, our understanding of the prognostic value of necroptosis-related genes with regulatory functions in CRC remains limited. Consequently, comprehensive and exhaustive analysis of the relationship between CRC progression and necroptosis is essential due to the complexity of necroptosis in intestinal tumorigenesis.

In this study, a risk-scoring estimation system was constructed based on genes related to necroptosis. Additionally, a nomogram was developed by integrating risk scores and clinical factors to enhance the model’s confidence in prognosis. The aim of this research is to evaluate the prognostic value of necroptosis-related genes (NRGs) in CRC and to establish a precise prediction instrument that can effectively assess the prognosis of CRC patients.

## Methods

### Data collection and preprocessing

Expression profiles of CRC tissues from GSE39582, GSE38832, and GSE161158 datasets were downloaded from the Gene Expression Omnibus (GEO) database, while the Cancer Genome Atlas (TCGA)- Colon Adenocarcinoma (COAD) transcriptome cohort data containing clinical data for 279 CRC samples were obtained from the UCSC Xena website. The GSE39582 dataset, comprising 518 CRC samples, served as the training cohort for constructing the prognosis prediction model, followed by external validation in three independent cohorts, including TCGA, GSE38832, and GSE161158. The microarray data from GSE17537, GSE17536, GSE38832, GSE16158, and GSE39582 were normalized using the “normalizeBetweenArrays” function in the limma package. For the RNA-seq data from the TCGA database, gene effective lengths were calculated using the R package GenomicFeatures, and count data were converted to TPM values, ensuring sample normalization. The databases used in the study were listed in **Supplementary Table S1**.

### Sequence download and OTU annotation

The rawdata were downloaded from peoject(project number:PRJNA995580), after downloaded the data, fastp software (version 0.19.4) was used to filter the rawdata. The filtered data was analyzed using QIIME2 software (version qiime2-amplicon-2024.2). Firstly, the cutadapt plugin was used to remove the sequencing primer sequences 338F (5′- ACTCCTACGGGAGGCAGCAG-3′) and 806R (5′- GACTACHVGGGTWTCTAAT-3′), then, vsearch was used for double ended merging, and the deblur denoise-16S differential was used to generate feature tables and representative sequences. The feature classifier classify sklarn plugin was used for object classification annotation.

### Microbiota and function difference

Alpha diversity and beta diversity was calculated and statistic using MicrobiotaProcess packages (version 1.19.0), boxplots were used to display alpha diversity differences between groups, PCoa was used to display the beta diversity among samples and groups. Picrust2 software was used to get functional enrichment analysis on microorganisms, and ggPicrust2 software was used for differential statistical analysis and visualization of KO results.

### Single sample gene set enrichment analysis

The R package “GSVA” was utilized to conduct single sample gene set enrichment analysis (ssGSEA) (22). The ssGSEA was applied to explore the enrichment of tumor-related pathways and immune cell infiltration in the GSE39582 database. Tumor-related datasets were acquired from hallmark gene sets in the MSigDB database (23). The characteristic gene set containing 28 immune cell types was obtained from a recent publication (Charoentong et al., 2017).

### Weighted gene co-expression networks analysis

WGCNA was carried out using the top 25% most differentially expressed genes in the GSE39582 dataset (24). Among all the soft threshold values, we selected the β value with the highest mean connectivity (β = 4). A minimum of 30 genes were set for high reliability of the results. Genes with a p-value of less than 0.001 were retained for further quantification of necroptosis-related genes and modules.

### Gene set enrichment analysis

The function of necroptosis-related genes was explored using GSEA (25). Separate chip expression profiles and sample data files were created for the training cohort and all validation cohorts, which were then imported into the GSEA software. Significant findings were considered at p <.05 and FDR p <.25 (26).

### Establishment and validation of a CRC prognostic predictive signature

Univariate Cox regression analysis was conducted to identify recurrence-free survival (RFS) and OS related cancer hallmarks. least absolute shrinkage and selection operator (LASSO) penalized Cox regression analysis was then applied to select necroptosis-related genes associated with prognosis. Finally, the LASSO Cox regression model was used to identify highly correlated necroptosis-related genes and construct the prognostic NRGs (27). The score of NGSs for each patient was calculated using the coefficients of Logistic Regression with the formula: NRGs score = ∑ (coefficient × mRNA expression) (28).

### Construction of nomogram for CRC prognosis prediction

Necroptosis scores and relevant clinical parameters were utilized to construct a nomogram using the “survival” and “rms” packages of R (29). The nomogram was designed to estimate 1-, 3-, and 5-year survival probabilities. The performance of the model was evaluated using the calibration curve and C index (30).

### Stromal and immune cells infiltration

We utilized xCell to estimate the cellular composition of stromal cells and immune cells within the tumor in the GSE39582 dataset (31). Subsequently, immune and stromal cell scores were calculated for each sample. To evaluate the infiltration of 22 immune cells in each sample, we employed the CIBERSORTx online website (32) Furthermore, the absolute abundance of eight immune cell populations of tissue-infiltrating immune cells was determined using the R package “MCPcount” (33).

### Drug sensitivity analysis

For drug sensitivity analysis, we employed the GSCALite database (34). Spearman correlation analysis was utilized to examine the relationship between the expression levels of 18 target genes and drug sensitivity.

### Cell lines and cell culture

The human CRC cell lines (HCT116, DLD-1, and NCM460) were procured from the American Type Culture Collection (ATCC). All cell lines were maintained in DMEM with 10% fetal bovine serum (HYCEZMBIO) in a humidified cell incubator at 37°C with 5% CO_2_.

### Reverse transcription reaction, quantitative reverse transcription polymerase chain reaction

Total RNA was extracted using Trizol (Takara Shuzo Co. Ltd, Kyoto, Japan) according to the manufacturer’s recommendations. Complementary DNA (cDNA) synthesis was performed using M-MLV reverse transcriptase (Thermo Scientific, Hudson, NH). Quantitative RT–PCR was conducted using the SYBR Green PCR kit (Takara Shuzo Co. Ltd, Kyoto, Japan) with the Real-Time PCR system (Applied Biosystems7500, Foster City, CA). The expression levels of mRNA were calculated using the 2^−ΔΔCt^ method. The primer sequences used in the study were listed in **Supplementary Table S2**.

### Single-cell RNA-seq data analysis

ScRNA-seq datasets (GSE132465) of human CRC were downloaded from the GEO database. The raw gene expression matrix was converted into Seurat objects by the Seurat R (version 4.1.0) package. To exclude low-quality cells, we filtered cells that expressed between 200 and 6000 genes, and <20% of mitochondrial gene expressions for further analysis. We standardized the data using the “PrepSCTIntegration” function. With FindIntegrationAnchors function of R package Seurat, Top 2000 high variable genes were used to create anchors. The “FindClusters” function with the “resolution” parameter set to 0.5. After batch effect correction, the cells were clustered into different subgroups by t-SNE projection, and cell types were annotated by SingleR (version 1.8.1). Subsequently, the expression of necroptosis-related genes in different cell types was analyzed.

### Statistical analysis

Data were analyzed using the R software. For forest plots, the hazard ratio (HR) was generated by univariate Cox or multivariate Cox proportional hazard regression. The Kaplan-Meier approach was used for survival analysis. Differences between groups were assessed using the Wilcoxon test. Statistically significant differences were indicated as follows: *p <.05; **p <.01; ***p <.001; NS, not significant.

## Results

### Identification of necroptosis as a primary risk factor for CRC prognosis

**Figure 1:**
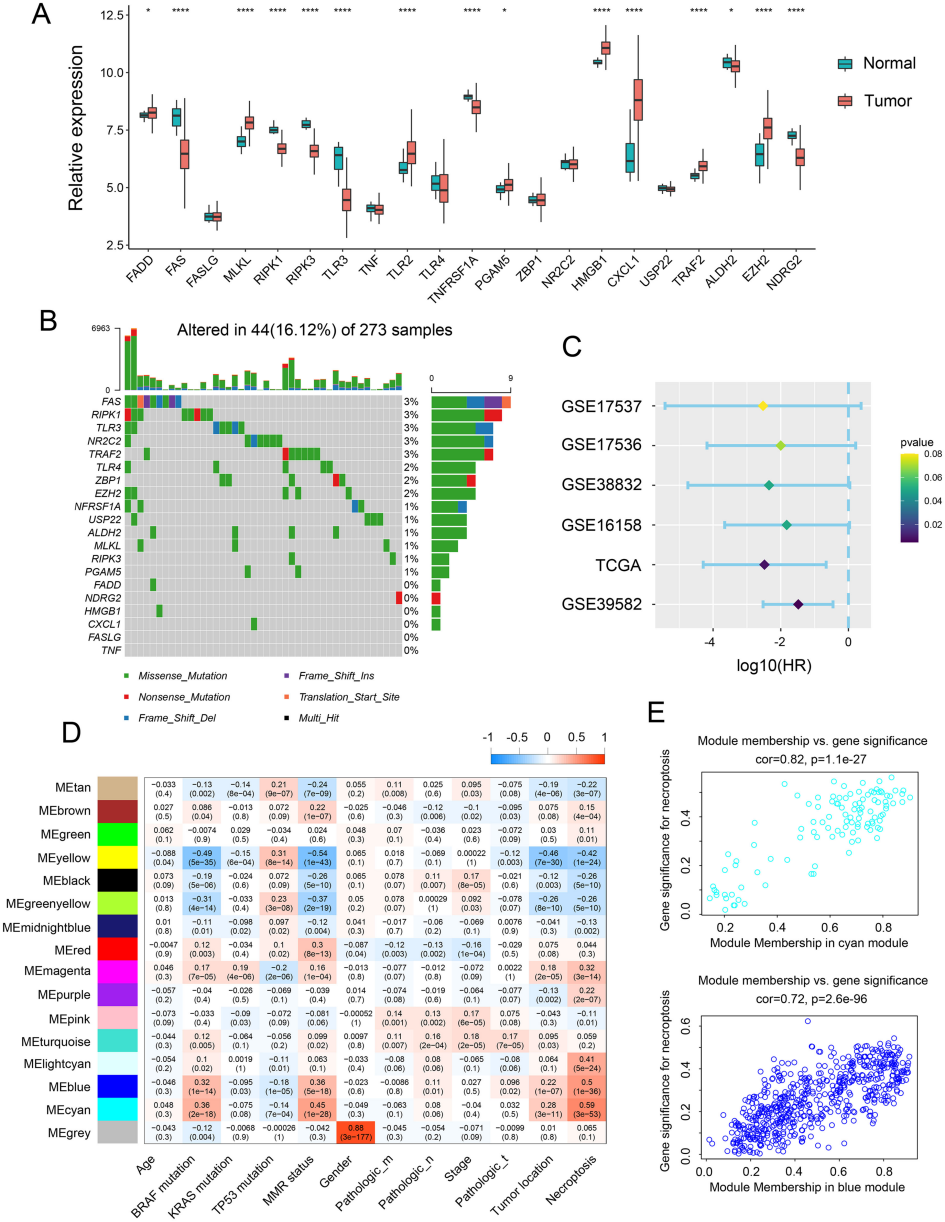
Expression landscape of necroptosis genes across CRC samples. **(A)** Transcript levels of 21 necroptosis genes in both colorectal normal and tumor tissues. **(B)** Landscape of genomic aberrations of necroptosis genes. **(C)** Forest plot depicting the hazard ratio of necroptosis genes for TCGA and GSE databases. **(D)** Heatmap illustrating the correlation between modules and clinicopathological characteristics. **(E)** Correlation between the cyan module & the blue module and gene significance for necroptosis. *p <.05, ****p <0.0001.

The relative expression of necroptosis genes (NGs) in tumor tissue and normal adjacent tissue were investigated to determine the influence of necroptosis on CRC development. Notably, mRNA expression analysis of 15 NGs revealed significant alterations. Further assessment demonstrated that the expression of necroptosis-related genes, including FADD, MLKL, TLR2, PGAM, HMGB1, CXCL 1, TRAF2, and EZH2, was elevated in tumor tissue, while FAS, RIPK1, RIPK3, TLR3, TNFRSF, ALDH2, and NDRG2 were upregulated in normal intestinal tissues (**Figure 1A**). Additionally, we analyzed genomic alterations among NGs in the TCGA CRC cohort, unveiling mutations occurring in 44 CRC patients, with missense mutation being the most prevalent type. Specifically, FAS, RIPK1, TLR3, NR2C2, and TRAF2 exhibited the highest frequency of mutations (**Figure 1B**). Furthermore, the prognostic value of these NGs was evaluated across various databases, including GSE39582, GSE17537, GSE17536, GSE16158, and TCGA. The analysis indicated a positive correlation between NGs and the prognosis of CRC patients (**Figure 1C**). To verify the clinical significance of NGs, we conducted WGCNA, comparing co-expression patterns in necroptosis with whole-transcriptome profiling data. Subsequently, MEblue and MEcyan modules, strongly associated with necroptosis, were selected for further study (**Figures 1D, E**).

### Construction of NRG signature

**Figure 2:**
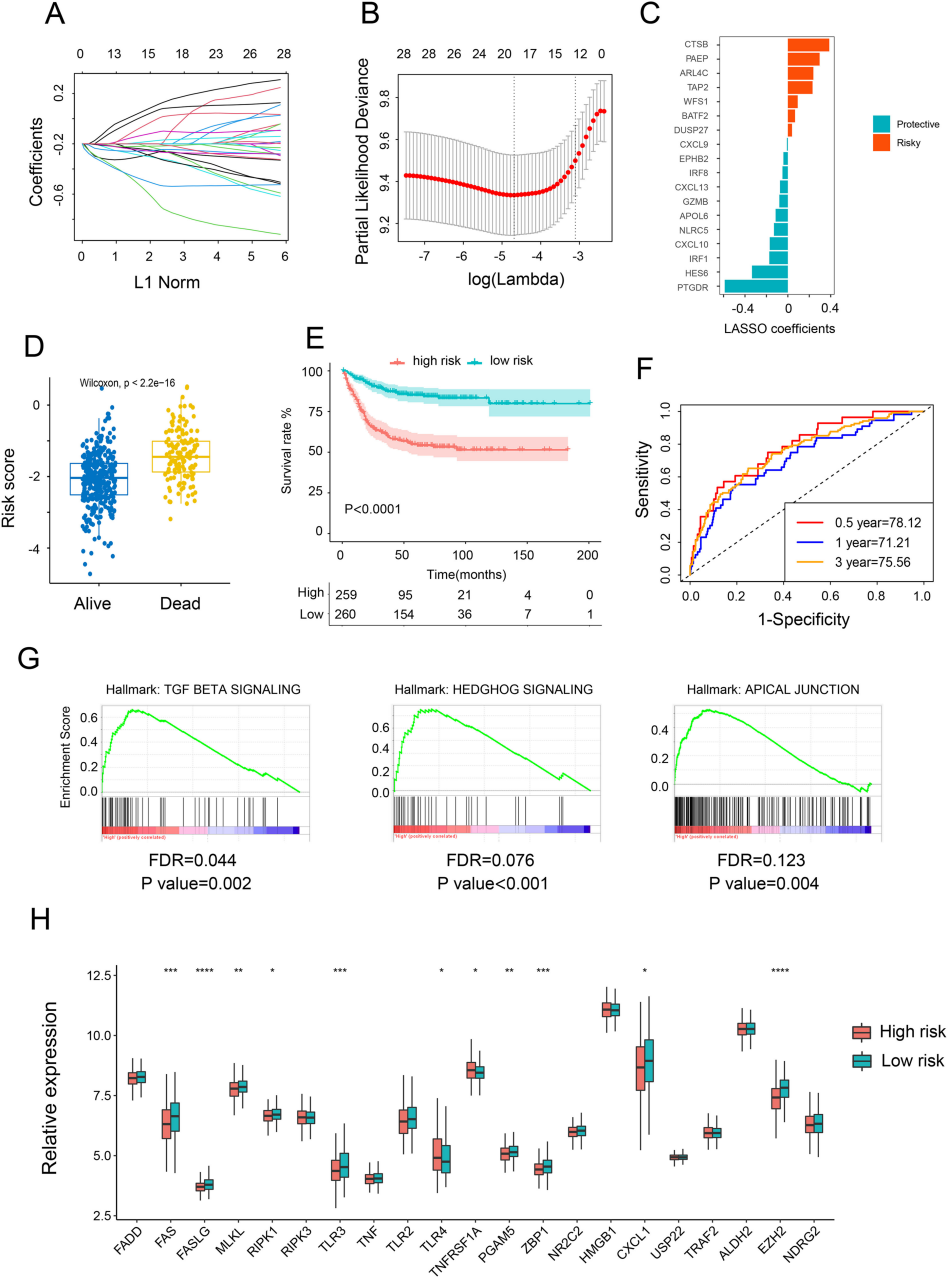
Construction and validation of risk score model. **(A)** Ten-fold cross-validation for variable selection in the LASSO model. **(B, C)** LASSO coefficient profiles of the 18 necroptosis-related genes. **(D)** Comparison of the risk scores of patients who are alive versus deceased based on the NRG model. **(E)** Kaplan–Meier OS curves for patients with high and low necroptosis scores. **(F)** Time-dependent ROC curves at 1, 3, and 5 years. **(G)** GSEA investigating biological pathways associated with necroptosis-high and necroptosis-low groups. **(H)** Relative mRNA expression of 21 necroptosis genes in patients with high and low necroptosis scores. *p <.05; **p <.01; ***p <.001, ****p <0.0001.

Initially, we applied LASSO Cox regression analysis to identify the most effective prognostic biomarkers within these modules. This process led to the identification of CTSB, PAEP, ARL4C, TAP2, WFS1, BATF2, DUSP29, CXCL9, EPHB2, IRF 8, CXCL13, GZMB, APOL6, NLRC5, CXCL10, IRF1, HES6, and PTGDR, which constituted the necroptosis-related genes (NRGs) risk model (**Figures 2A–C**). Subsequently, we estimated the NRG score for each individual by integrating the expression of these 18 NRGs. Patients were then categorized as either NRG-high or NRG-low based on the median NRG score. To discern the characteristics of the NRGs high and low-risk groups, we conducted Cox regression analysis comparing clinical factors such as age, gender, TNM stages, and location. The findings revealed that the high-risk group exhibited advanced stages and higher risk. Additionally, the high-risk NRG score was associated with higher mortality in survival analysis (**Figure 2D**) and correlated with adverse outcomes in Kaplan-Meier analysis (**Figure 2E**). These findings were further validated to establish the reproducibility and validity of the NRG signature. With respective AUC values of 78.12%, 71.21%, and 75.56%, the gene signature effectively predicted the 0.5-year, 1-year, and 3-year OS rates of CRC patients, demonstrating a significantly robust predictive performance (**Figures 2F**). These results were reaffirmed across three other independent datasets (**Supplementary Figure S2A–I**). Furthermore, GSEA demonstrated a pronounced association between NRG-high group genes and markers of tumor-promoted progression, such as TGF BETA SIGNALING, HEDGHOG SIGNALING, and APICAL JUNCTION (**Figure 2G**). Upon reanalyzing the NGs, it became evident that most NGs were upregulated in the low-risk group (**Figure 2H**), consistent with our earlier conclusion regarding the positive correlation between NGs and the prognosis of CRC patients.

### Re-validation of the prognostic significance of NRGs through independent cohorts

**Figure 3:**
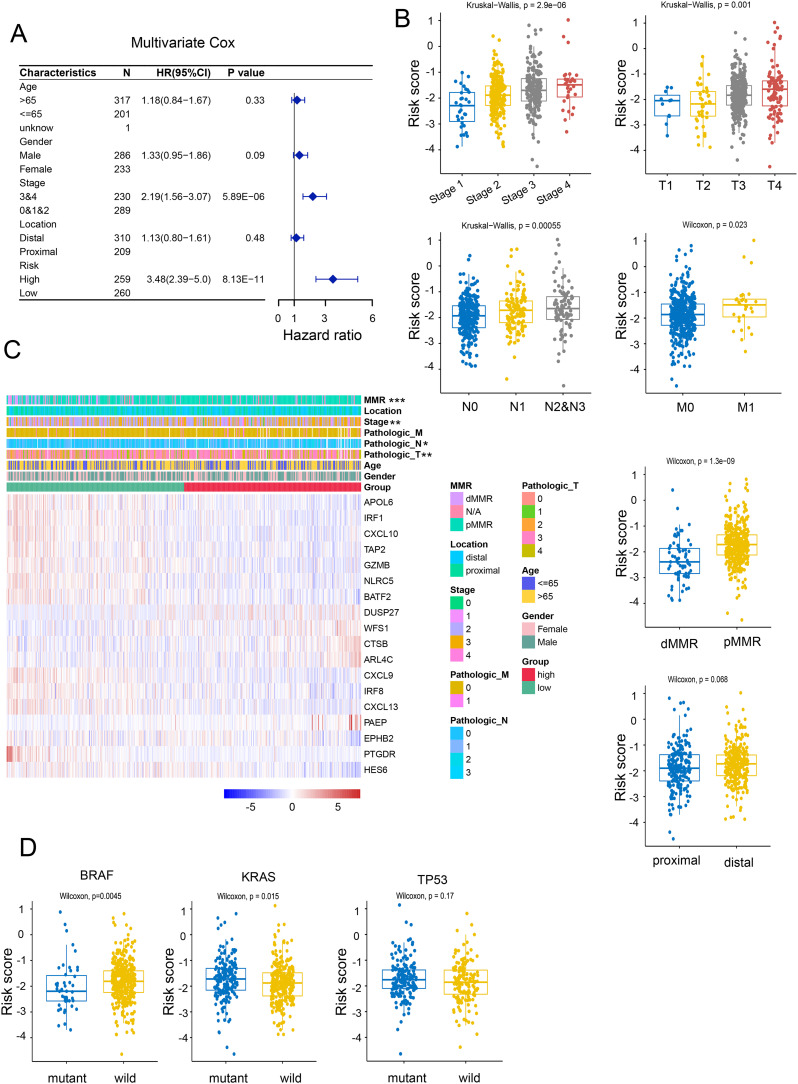
Correlation of risk models with clinical characteristics. **(A)** Univariate Cox regression analyses of necroptosis-related scores and other clinicopathological factors. **(B)** Correlation of NRG with clinicopathological characteristics. **(C)** Relative expression of necroptosis-related genes and MMR status between necroptosis-high and necroptosis-low patients. **(D)** Correlation between BRAF, KRAS, p53 mutations and NRG. *p <.05; **p <.01; ***p <.001.

By utilizing multivariate and univariate Cox regression analyses, we further assessed the prognostic value of NRGs and other clinicopathological factors. The results suggested that these gene signatures may serve as valuable prognostic parameters independent of other clinical factors (**Figure 3A**). The heatmap analysis revealed a significant association between NRGs expression and clinicopathological parameters, including stage, tumor location, and gender (**Figure 3C**). Further investigation uncovered that the NRG-high group was associated with advanced clinical stage, worse clinicopathological grade, and a higher frequency of deficient DNA mismatch repair (DMR) (**Figure 3B**), along with a correlation with Kirsten rat sarcoma 2 viral oncogene homolog (KRAS) gene mutation and an anticorrelation with BRAF mutation, while no significant difference was observed in TP53 mutation (**Figure 3D**). These findings indicated that the necroptosis signature we proposed could be utilized to predict prognosis in additional colon cancer cohorts.

### Potential of NRG-based nomogram in survival prediction of CRC

**Figure 4:**
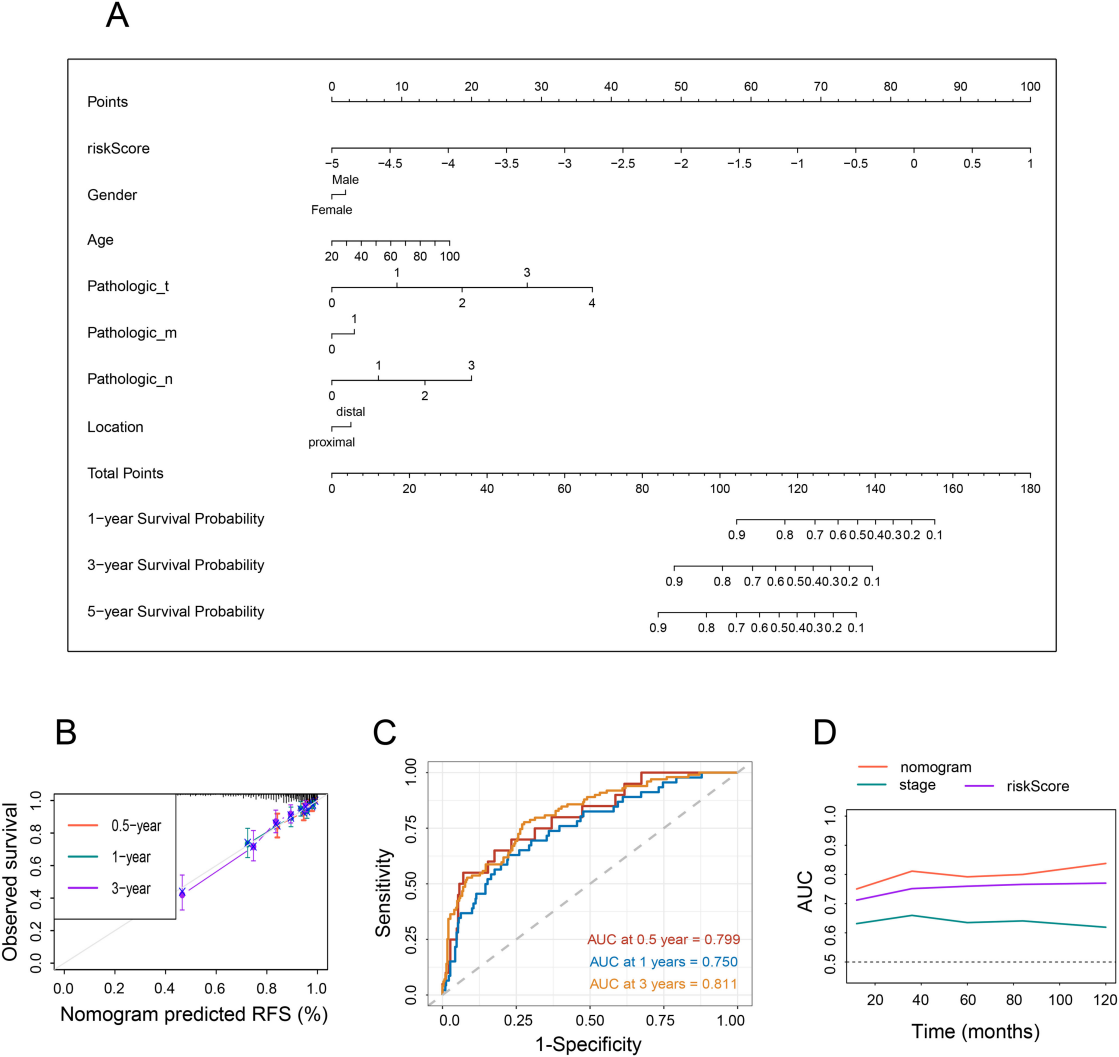
Nomogram and assessment of the risk model. **(A)** Development of a nomogram based on NRG and clinicopathological characteristics. **(B)** Calibration curves. **(C)** Time-dependent ROC curves at 1, 3, and 5 years. **(D)** Line graph illustrating the area under the curve.

By integrating NRG with clinicopathological traits (sex, age, location, pathologic AJCC stages) of the individuals, a novel nomogram was created to predict the OS probability of CRC patients (**Figure 4A**). The nomogram exhibited a high degree of consistency between observed and predicted survival probabilities (**Figure 4B**). Additionally, the areas under the ROC were 0.799, 0.750, and 0.811, respectively, for the nomogram’s 1-, 3-, and 5-year OS (**Figures 4C, D**), indicating that the nomogram had a superior capacity for OS prediction compared to other risk models. These findings demonstrate that the newly developed nomogram can accurately predict CRC prognosis.

### Correlation between risk score and immunotherapy response

**Figure 5:**
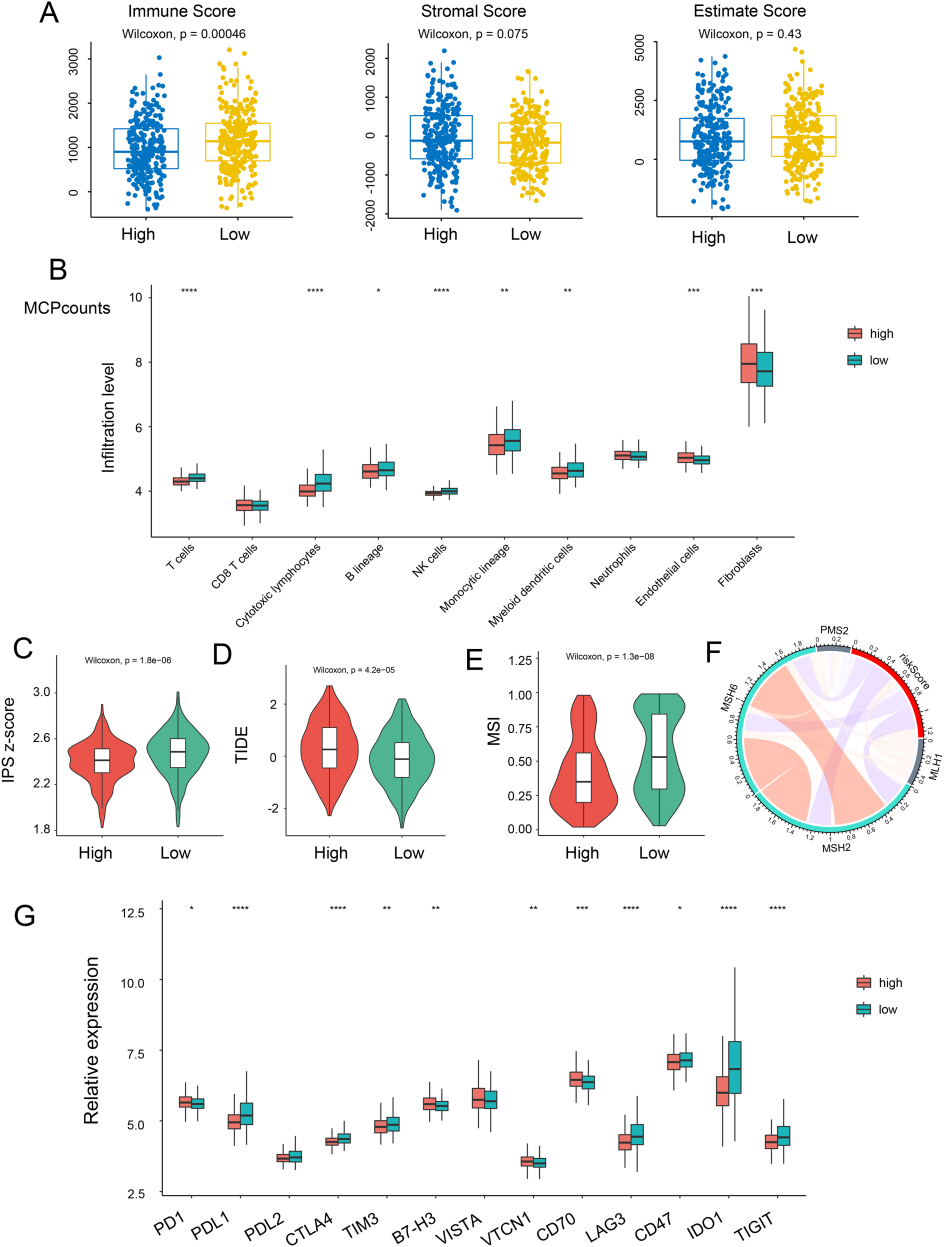
Relationship between risk score and immune status. **(A)** The correlation of immune, stromal, and estimate scores with NRG-high and NRG-low patients. **(B)** Relative infiltration levels of immune cells in NRG-high risk and NRG-low risk. Violin plot representing the IPS **(C)**, TIDE **(D)**, and MSI **(E)** scores between NRG-high and NRG-low groups. **(F)** Correlation between risk score and MLH1, MSH2, MSH6, and PMS2. **(G)** Gene expression of immune checkpoints between the two NRG-high and NRG-low groups. *p <.05; **p <.01; ***p <.001, ****p <0.0001.

Since the TME plays a crucial role in cancer development and treatment, we explored the variances in immune characteristics among the different NRS-score groups. The results indicated that immune signatures, such as immune and stromal scores, exhibited a negative correlation with the NRG score (**Figure 5A**). A correlation matrix between NRG and TME cells, encompassing immune cells and stromal cells, revealed that T cells, cytotoxic lymphocytes, NK cells, and monocytic lineage were significantly more abundant in the NRG-low group, while endothelial cells and fibroblasts were notably lower in the NRG-low group (**Figure 5B**, **Supplementary Figures S3A, B**). In The Cancer Immunome Atlas, the immunogenicity-based Immune Prognostic Score (IPS) was capable of predicting patients’ responses to immunotherapy with high accuracy. Subsequently, we investigated the relationship between IPS and NRG risk score. Surprisingly, we discovered that the total IPS of the NRG low-risk group was remarkably higher than that of the high-risk group, suggesting a superior response to immunotherapy in individuals with lower-risk scores (**Figure 5C**). Tumor Immune Dysfunction and Exclusion (TIDE) scores were then calculated to predict immunotherapy response. Similarly, patients in the low-risk group had lower TIDE scores, indicating a more favorable immune response (**Figure 5D**). Furthermore, microsatellite instability (MSI) genes such as MSH2, MSH6, MLH1, PMS2—used to evaluate responses to immunotherapy—were found to be upregulated in the lower-score group, suggesting potential benefits from immunotherapy strategies (**Figures 5E, F**). Additionally, we evaluated the expression of immune checkpoint-related genes and observed an increase in PDL1, PDL2, CTLA4, TIM3, IDO1, and TIGIT in the NRG low-score group (**Figure 5G**).

The Major Histocompatibility Complex (MHC), which plays a pivotal role in tumor immunological effects by presenting peptides recognized by cytolytic T cells, was the subject of our investigation to ascertain a risk score’s potential for predicting immune response. We conducted an analysis of 20 MHC antigens across the two risk groups and found that 14 histocompatibility antigens (HLA)-related antigens were upregulated in patients with NRG low-risk scores (**Figure 4SA**). This suggests that individuals with lower scores may benefit from immunotherapy. In summary, our necroptosis signature could serve as a dependable predictor of the immune response.

### Analysis of genome mutation and drug susceptibility

**Figure 6:**
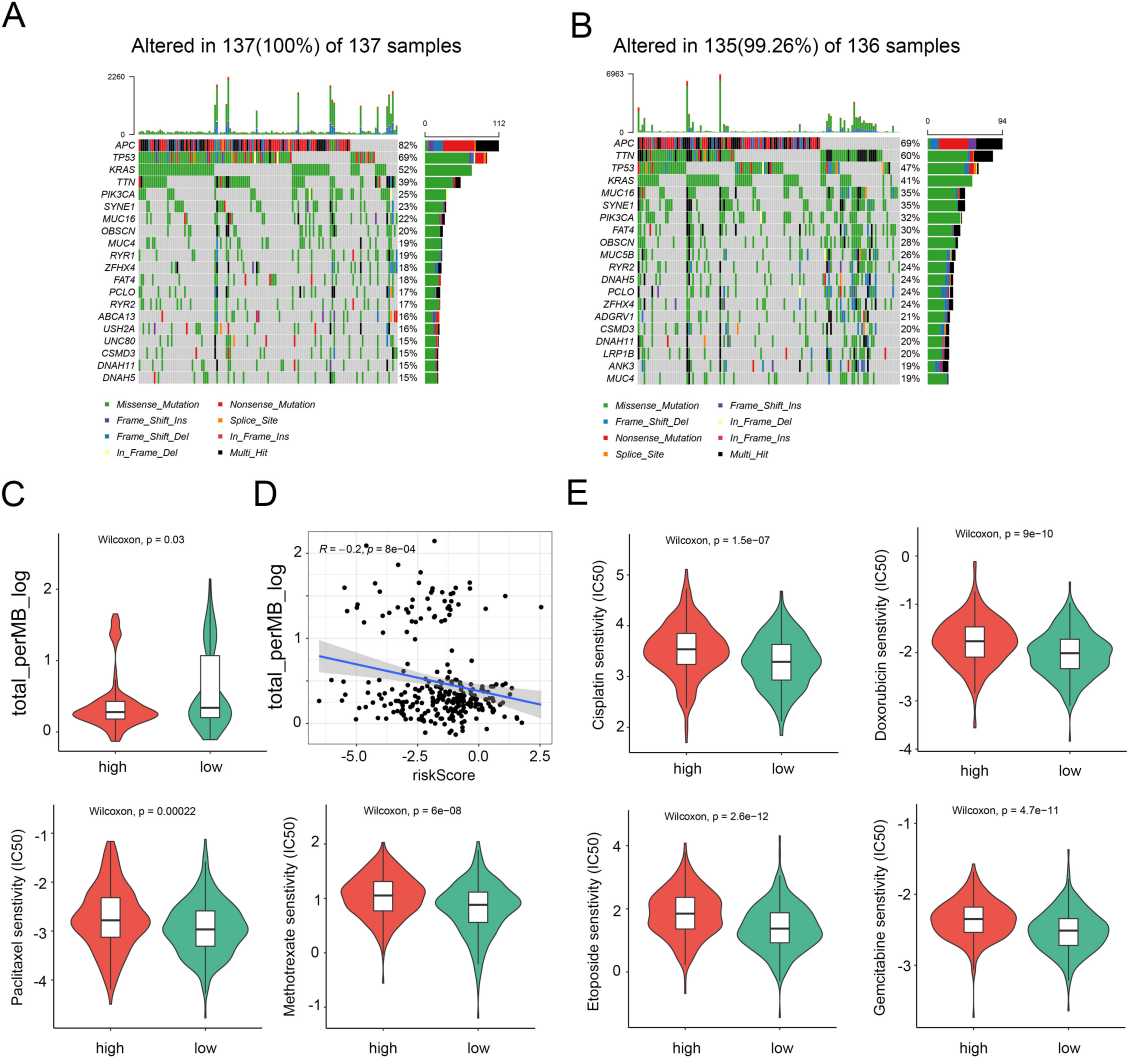
Distinction between high and low NRG risk groups and immunotherapy prediction. **(A)** Landscape of genomic mutations in the NRG-high group. **(B)** Landscape of genomic aberrations in the NRG-low group. **(C)** TMB score in the two risk groups. **(D)** Correlation between TMB score and risk score. **(E)** Six common therapeutic drugs with differential IC50 between the two NRG groups.

In light of the significance of genomic changes in CRC development, we conducted an investigation into genetic mutations between the two groups. According to the mutation map, the top three mutational genes in both groups were TP53, APC, and TTN (**Figures 6A, B**). Substantial evidence indicates that individuals with a high Tumor Mutational Burden (TMB) could benefit from immunological therapy due to the abundance of neoantigens. The TMB observed in the high-risk group was lower than that in the low-risk group, suggesting potential benefits from immunotherapy for the low-risk group (**Figure 6C**). Furthermore, Spearman correlation analysis revealed an inverse relationship between the NRG score and the TMB (**Figure 6D**). Subsequently, we evaluated the drug sensitivity of individuals in the NRG low and high-risk groups using chemotherapeutic medicines currently employed for the treatment of CRC. It was found that individuals with a low NRG score exhibited lower C50 values for chemotherapeutics, such as cisplatin, doxorubicin, gemcitabine, paclitaxel, Methotrexate, etoposide, and etoposide (**Figure 6E**). Collectively, these findings suggest that NRGs are associated with drug sensitivity.

### Validation of NRG in colon cancer cell lines and single-cell level

**Figure 7:**
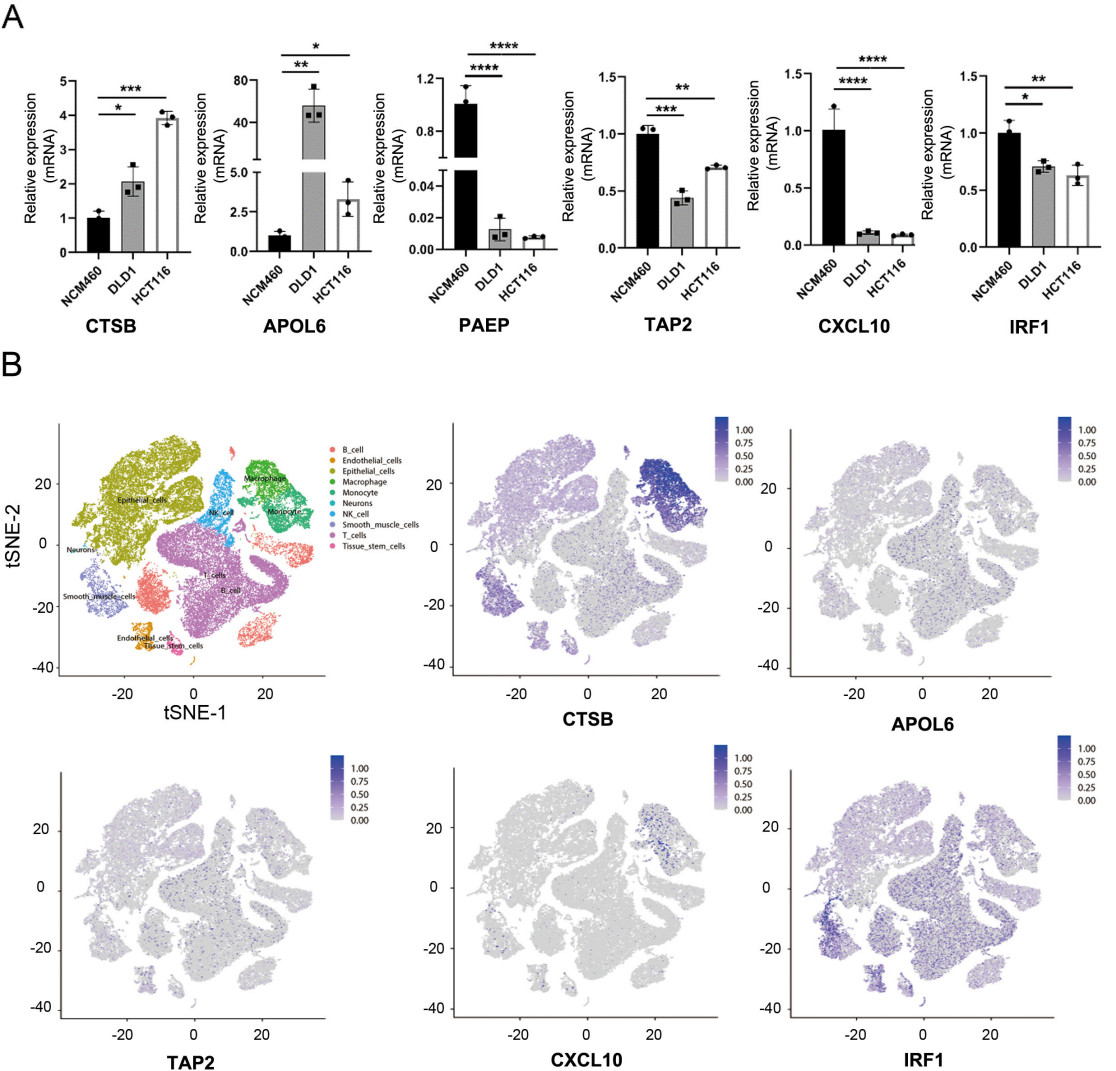
Validation of necroptosis-related genes. **(A)** mRNA expression levels of CTSB, APOL6, PAEP, TAP2, CXCL10, and IRF1 in various colon cancer cell lines. **(B)** t-SNE plot revealing single-cell transcriptomic profiling of CTSB, APOLA6, TAP2, CXCL10, and IRF1 from CRC. *p <.05; **p <.01; ***p <.001, ****p <0.0001.

To validate our data analysis, we assessed NGR mRNA expression levels in two CRC cell lines (DLD1 and HCT116) and a normal epithelial colon cell line (NCM460). As anticipated, the expression of the risk gene CSTB in colon cancer cells was significantly higher than in NCM460 cells. Conversely, protective genes such as PAEP, TAP2, CXCL10, and IRF1 were notably down-regulated in colon cancer cells (**Figures 7A**, **S5A**). However, it’s worth noting that the risk gene, APOL6, was unexpectedly and dramatically increased in CRC, and other NGR expression levels did not exhibit significant differences (**Figure 7A**). One possible reason for this could be the limited number of cell lines that were analyzed. At the single-cell level, CTSB and CXCL10 were primarily expressed in macrophages and monocytes. APOLA6 and TAP2 were predominantly expressed in NK cells, T cells, B cells, macrophages, and monocytes. IRF1 was mainly expressed in smooth muscle cells, endothelial cells, tissue stem cells, T cells, and B cells, indicating that these cell types may be major contributors to necroptosis in CRC (**Figures 7B**, **S5B**).

### Intratumoral microorganisms facilitates tumor necroptosis

**Figure 8:**
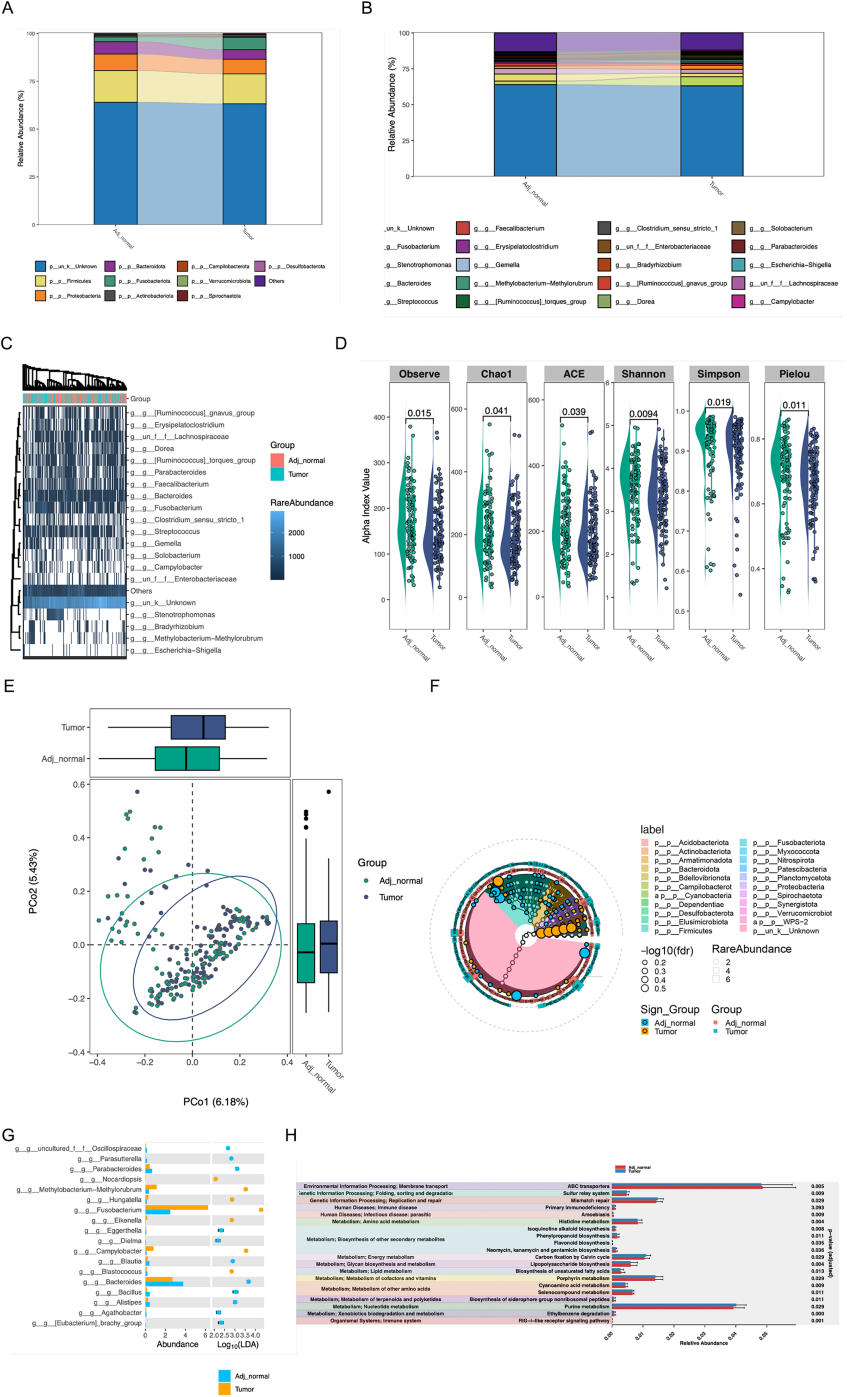
The effect of intratumoral Microorganisms on the progression of Tumor Necroptosis **(A, B)** The Sankey diagram showed the microbiota composition at phylum and genus levels. **(C)** The heatmap of microbial at the genus level, the abundance information of the top 20 microorganisms were displayed. **(D, E)** The alpha diversity and beta diversity between the tumor and adjacent normal groups. **(F)** The biomarker analysis of aquatic microorganisms was presented in cladogram format. **(G)** the analysis result of LDA between the tumor and adjacent normal groups were shown **(H)** The analysis of functional differences in microorganisms between the tumor and adjacent normal groups were presented.

We subsequently analyzed the microorganisms in colorectal tumor tissues and adjacent normal tissues, the microbiota composition at phylum and genus levels was shown by the Sankey plot of microbial species, which revealed that the microorganisms of adjacent normal tissue and intratumor were remarkably distinct (**Figures 8A, B**). Alpha and beta diversity showed significant difference between tumor and adjacent tumor groups (**Figures 8D, E**). Although the heatmap demonstrated that the variety of microorganisms in both colorectal tumor tissues and adjacent normal tissues were basically consistent, including Erysipelatoclostridium, Faecalibacterium, Stenotrophomonas, the abundance of microorganisms in colorectal tumor tissues and normal tissues was significantly different (**Figures 8C, F, G**), for instance, Faecalibacterium which is considered to secret lipopolysaccharide to subsequently activate necroptosis, was outstandingly upregulated in intratumor. In addition, microbial functional enrichment analysis revealed mismatch repair and purine metabolism signaling pathway is higher in tumor group than in control group (**Figure 8H**).

## Discussion

CRC poses a significant threat to global health due to its high morbidity and mortality rates. Necroptosis is closely associated with tumor pathogenesis and progression, and is considered a potential vulnerability of tumors that could be targeted for anti-tumor therapy. However, the influence of NRGs on CRC remains unclear. Necroptosis appears to be a favorable factor in CRC, as downregulation of several necroptosis related molecules were associated with poor prognosis in CRC (20). While, another study reported that RIPK3 had positively impact on inflammation-associated CRC development in mice models (35). Further studies are required to better understand these conflicting observations. In this study, the relationship between the NRGs and CRC was systematically investigated by bioinformatics. We uncovered that necroptosis may serve as a primary risk factor for a better prognosis in CRC patients. Furthermore, a risk model based on 18 necroptosis-related genes was constructed and subsequently validated across independent CRC cohorts. In addition, we developed a CRC prediction nomogram by integrating the expression profiles of NRGs with the clinicopathological features of CRC patients. Within this framework, we identified two distinct necroptosis-related groups, designated as the high-risk group and the low-risk group based on the expression of NRGs. Our findings revealed that individuals in the high-risk group exhibit a poorer prognosis, stronger immune suppression, and lower responsiveness to immunotherapy.

Necroptosis has been noted to trigger a robust immune response. Both CTLs and NK cells are subtypes of immune cells capable of eliminating tumor cells through cytotoxic mechanisms. Substantial evidence indicates that tumor-infiltrating CTLs and NK cells are correlated with the prognosis and outcome of CRC patients. Reduced NK cell infiltration or impaired NK cell functions have been linked to poor overall patient survival and a higher relapse rate of CRC after treatment (36–38).Moreover, RIP3, a central adaptor of receptor-interacting protein kinase 3, has been shown to accelerate the progression of colitis-associated CRC by suppressing the infiltration of cytotoxic lymphocytes (35). Inhibition of critical necroptosis modulators RIP1, RIP3, and MLKL significantly impairs necrotic death, unveiling a novel cytotoxic pathway of NK cells via granzyme-induced necroptosis, which provides target selectivity (39). In our investigation, we explored the relationship between NRG score and immune infiltration. Our findings in this study revealed that the high-risk group exhibited notably lower immune scores and TIDE scores, signifying that low-risk patients were characterized as “immune hot,” with significantly higher immune checkpoint activity. Furthermore, we demonstrated that the lower-risk group had greater infiltration of antitumor cells, including T cells, cytotoxic lymphocytes, NK cells.

In addition to CTLs and NK cells, the monocytic lineage, which includes DCs and macrophages, plays a crucial role in anti-tumor immunity by regulating and enhancing the cytotoxic effects of NK cells. DCs, due to differences in their maturation status, location, and interaction with other tumor-infiltrating immune cells, have been reported to exert either a positive or negative influence on CRC prognosis. Notably, MLKL can activate RIPK3, a key downstream mediator, subsequently prompting the oligomerization of RIPK3 to impair membrane integrity, resulting in necroptotic cell death. In fact, MLKL has been shown to have a suppressive effect on the progression of intestinal tumorigenesis by reducing IL-6 production by dendritic cells (20). Additionally, another study demonstrated that Mlkl-/- mice promote azoxymethane-dextran sulphate-sodium (AOM/DSS)-induced colitis-tumorigenesis by suppressing ERK activation and cytokine expression in DCs (40). Our study revealed that DCs, and monocytic lineage were much greater infiltrated in NGS lower-risk group. These findings suggest that necroptosis-mediated signals play a role in DC-mediated tumor killing. However, the influence of necroptosis on CRC progression still requires further investigation.

The regulation of immune homeostasis in the TME is governed by immunological checkpoints, which control and modulate the timing and intensity of immune responses. However, receptor-based signal activation cascades mediated by immune checkpoints have a negative impact on T cell regulation, enabling tumor cells to evade immune surveillance by inducing immune tolerance. Immune checkpoint inhibitors prevent checkpoint proteins from interacting with their binding partners, thereby allowing activated T lymphocytes to eliminate cancer cells. Immune checkpoint inhibitor treatment has been recommended for various cancer types, including CRC. Within the TME, cancer cells evade immune attacks, and distinct immunological profiles predict different outcomes of immunotherapy. In our current study, we revealed that the IPS score, which predicts the immunotherapy response of patients, is higher in the necroptosis-lower subgroup, consistent with the downregulated TIDE score, indicating that the lower-risk subgroup is likely to have a better prognosis. Additionally, MSI genes were increased in the necroptosis lower risk subgroup. Indeed, we observed that the majority of immune checkpoints were overexpressed in the lower subtype of necroptosis; therefore, we assert that the lower-risk group will be more vulnerable to immune checkpoint inhibition.

MHC molecules, also known as major histocompatibility complexes, are cell-surface molecules that present antigens to T cells, allowing these cells to distinguish between normal and tumor cells. The loss of HLA can enable cancer cells to evade CTL lysis and immune surveillance. Our findings demonstrate that a majority of HLA were increased in the necroptosis low-risk group, suggesting that patients in the NRGs low-risk subgroup could benefit from immunotherapy. Despite this, the therapeutic efficacy of immunotherapy for CRC remains limited. Combination therapy plays a more significant role since single immunotherapy is not always suitable for individuals. Through our analysis of drug sensitivity, we found that the inhibitory concentration (IC50) of several anti-tumor drugs was lower in the NRGs-low group, suggesting that these drugs may be beneficial for the treatment of necroptosis low-risk patients. These drugs include cisplatin, doxorubicin, paclitaxel, methotrexate, etoposide, and gemcitabine. As a result, the model developed by us can better evaluate sensitivity to chemotherapy and immunotherapy in high and low-risk groups, thus enabling the potential integration of multiple treatment strategies in CRC, such as combining chemotherapy with immunotherapy.

Many researches devoted to investigate the overall role of certain classes of genes in CRC’s progression, such as hypoxia related genes, aging related genes and ferroptosis related genes (41–43). Classifying CRC patients into different subtypes based on necroptosis-related genes may help to clarify the roles of necroptosis in CRC, understand the heterogeneity of CRC patients and provide personalized treatment options. We have noticed that some studied focused on the influence of necroptosis-related genes on the progression of CRC. Wei and Xinyi’s research indicates that the low-risk group has a poorer prognosis, which were contrary to our conclusion. The CRC prediction nomogram established by Wei and Xinyi is only based on the TCGA database and has not been further validated by tumor biology experiments, besides that, the accuracy of prediction nomograms on the long-term forecasting were also lower compared with our NRG nomogram (44, 45). 18 NRGs, including CTSB, PAEP, ARL4C, TAP2, WFS1, BATF2, DUSP29, CXCL9, EPHB2, IRF8, CXCL13, GZMB, APOL6, NLRC5, CXCL10, IRF1, HES6, and PTGDR, were screened for our signature. Given that the conclusion of our research was based on bioinformatic analysis using multiple of public databases, prospective investigations are needed to explore the association between CRC clinical characteristics and necroptosis. However, experimental validation is required to elucidate the mechanisms underlying the impact of the NRGs on the TME of CRC.

Finally, we also analyzed the changes in gut microbiota in intestinal tumor tissues and normal tissues and found that significant difference of microorganisms on the activation of necroptosis was in existence from internal and adjacent tissues. These phenomena might provide new targets to eliminate the influence of intratumoral microorganisms.

## Conclusion

This study demonstrated the substantial relationship between necroptosis and colorectal cancer through bioinformatics analyses. It may be helpful in the prognostic improvement and clinical management of individuals with colorectal cancer. The necroptosis signature uncovered could be valuable in predicting prognosis among CRC patients and could aid in identifying therapeutic targets in colorectal cancer.

## Data Availability

The original contributions presented in the study are included in the article/**Supplementary Material**, further inquiries can be directed to the corresponding author/s.
