# Supplementary material for: Role of necroptosis-related genes in immune activity and prognosis of colorectal cancer
Source: Front Immunol. 2025 Sep 1;16:1619749. doi: 10.3389/fimmu.2025.1619749 (PMC12434056; doi:10.3389/fimmu.2025.1619749)
Supplement: Supplementary file 1 [file DataSheet1.docx]

**Necroptosis-Related Genes Associated with Immune Activity and Prognosis of Colorectal Cancer**

Lulu Tan^1, 2†^, Shuaifeng Wang ^5†^, Weilong Chang^1†^, Xiaoying Zhang^3^, Rui Deng^1^, Huifang Yan^6^, Weiwei Zhu^4, 5^, Huifen Wang^4, 5^, Yudie Cai^4, 5^, Zhibo Liu ^*1^, Yuyan Tan^*2^, Jinyuan Cui^*1^

*^1^Department of Gastrointestinal Surgery, the First Affiliated Hospital of Zhengzhou University, 450052, Zhengzhou, China*

*^2^Department of Breast and Thyroid Surgery, the First College of Clinical Medical Science, China Three Gorges University, Yichang,* *443000, China*

*^3^The First college of Clinical Medical Science, China Three Gorges University, Yichang,* *443000, China*

*^4^Department of Infectious Diseases, the First Affiliated Hospital of Zhengzhou University, 450052, Zhengzhou, China*

*^5^Gene Hospital of Henan Province, First Affiliated Hospital of Zhengzhou University, 450052, Zhengzhou, China*

*^6^Department of Radiotherapy, The Second Affiliated Hospital of Zhengzhou University, Zhengzhou, 450014, China*

^*^ Corresponding author.

*E-mail addresses*: [*lzl1041100271@163.com*](mailto:lzl1041100271@163.com) *(Zhibo Liu),* [*tyytyz@sina.com*](mailto:tyytyz@sina.com) *(Yuyan Tan),* *[jinyuan_c@163.com](mailto:jinyuan_c@163.com) (Jinyuan Cui).*

^†^These authors contributed equally to this work.


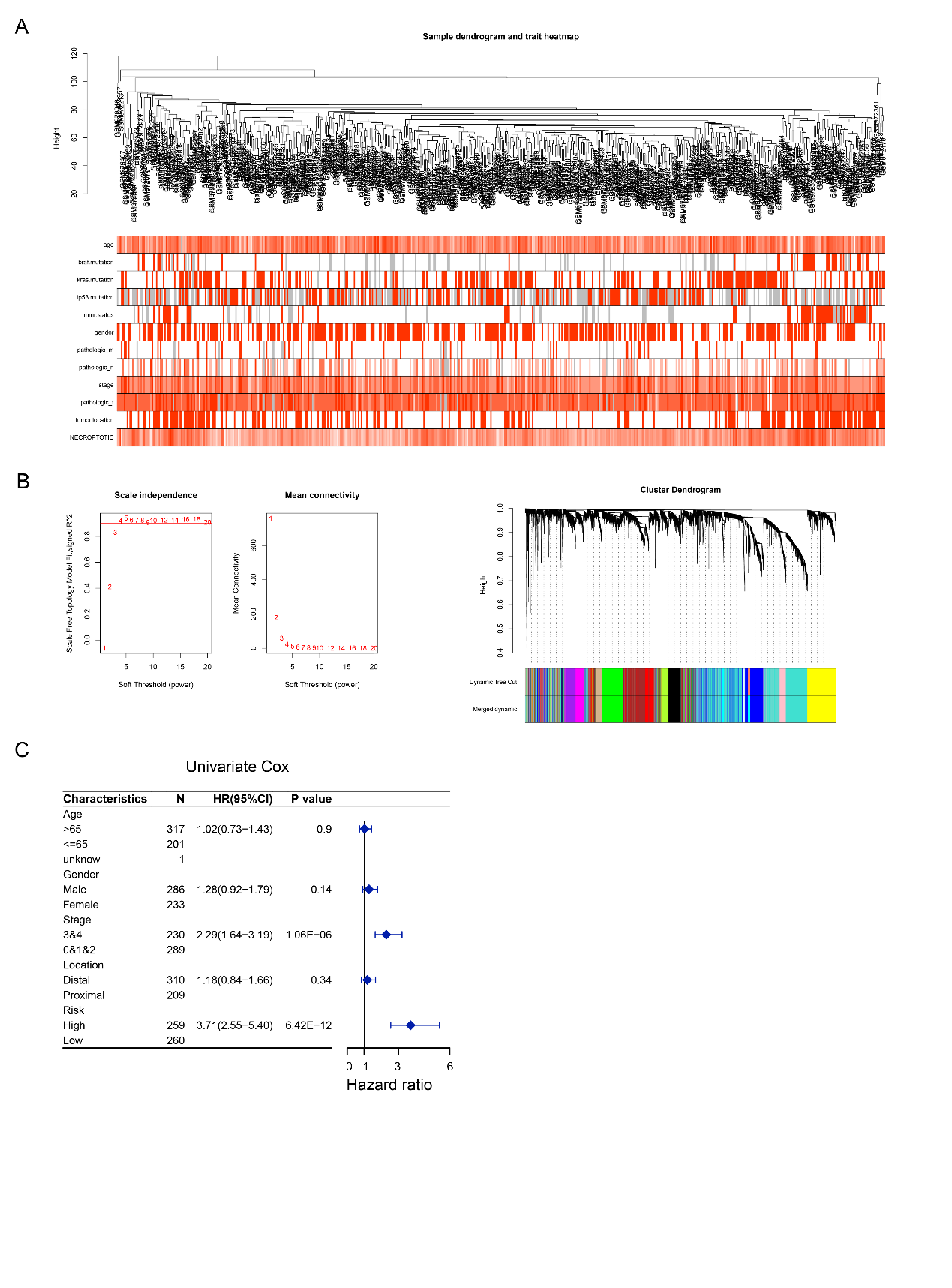
**FIGURE S1** WGCNA construction and gene significance for the clinical significance of necroptosis genes. (A) Clustering dendrogram of 203 samples from WGCNA. (B) Calculating the scale-free index for different soft-threshold powers (β) and the mean connectivity for different soft-threshold powers. (C) Univariate Cox regression analysis of necroptosis risk scrore and clinicopathological characteristics.


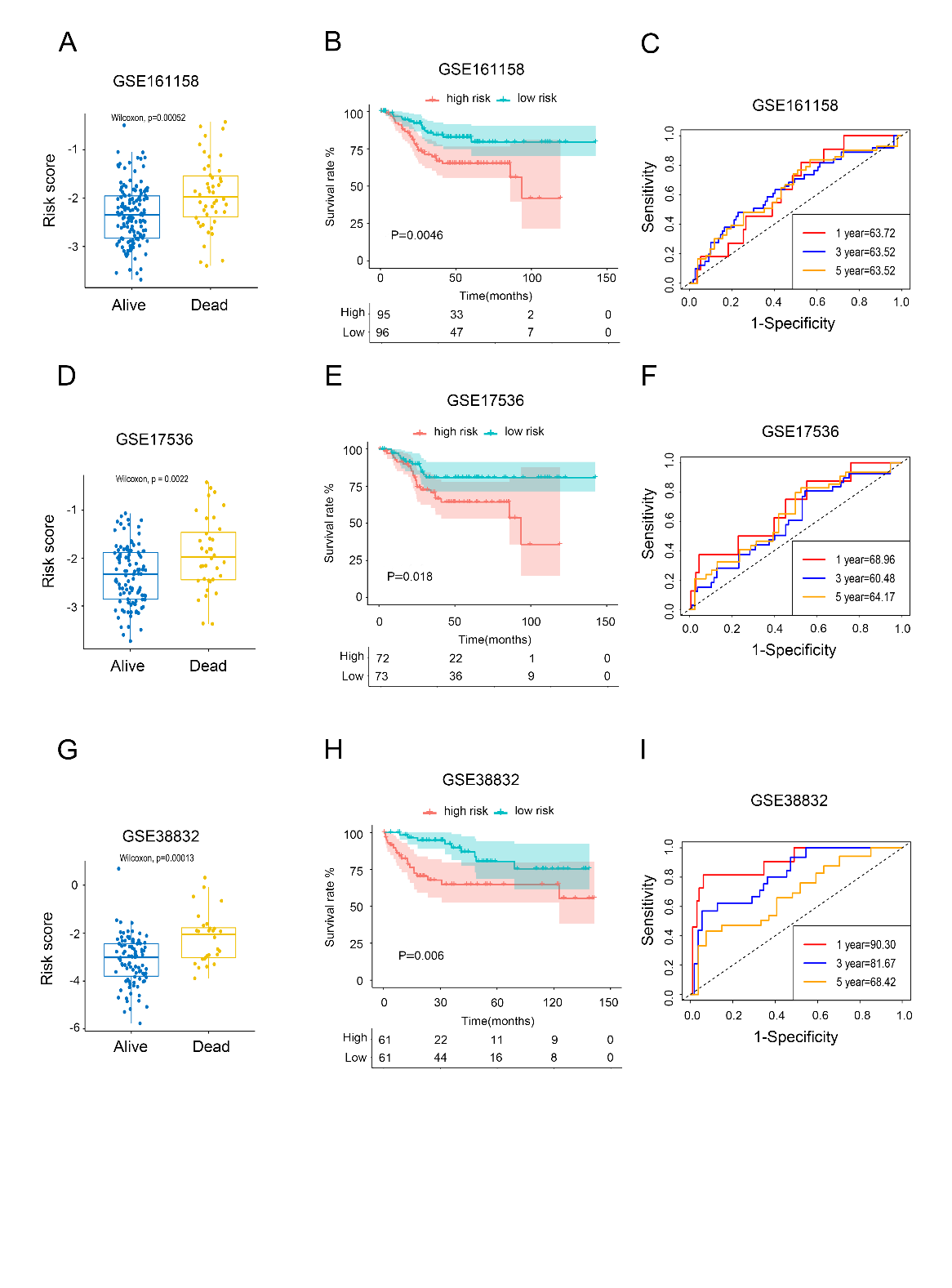


**FIGURE S2**. Correlation of risk models with clinical characteristics. Comparison of NGS scores in living and dead CRC patients in GSE161158 (A). Kaplan–Meier OS curves for CRC patients in NGS-high and -low group in GSE161158 (B). Time-dependent ROC curves at 0.5, 1 and 3 years in GSE161158(C). Comparison of NGS scores in living and dead CRC patients in GSE17536 (D). Kaplan–Meier OS curves for CRC patients in NGS-high and -low group in GSE17536(E). Time-dependent ROC curves at 0.5, 1 and 3 years in GSE17536 (F). Comparison of NGS scores in living and dead CRC patients in GSE38832 (G). Kaplan–Meier OS curves for CRC patients in NGS-high and -low group in GSE38832(H). Time-dependent ROC curves at 0.5, 1 and 3 years in GSE38832 (I).


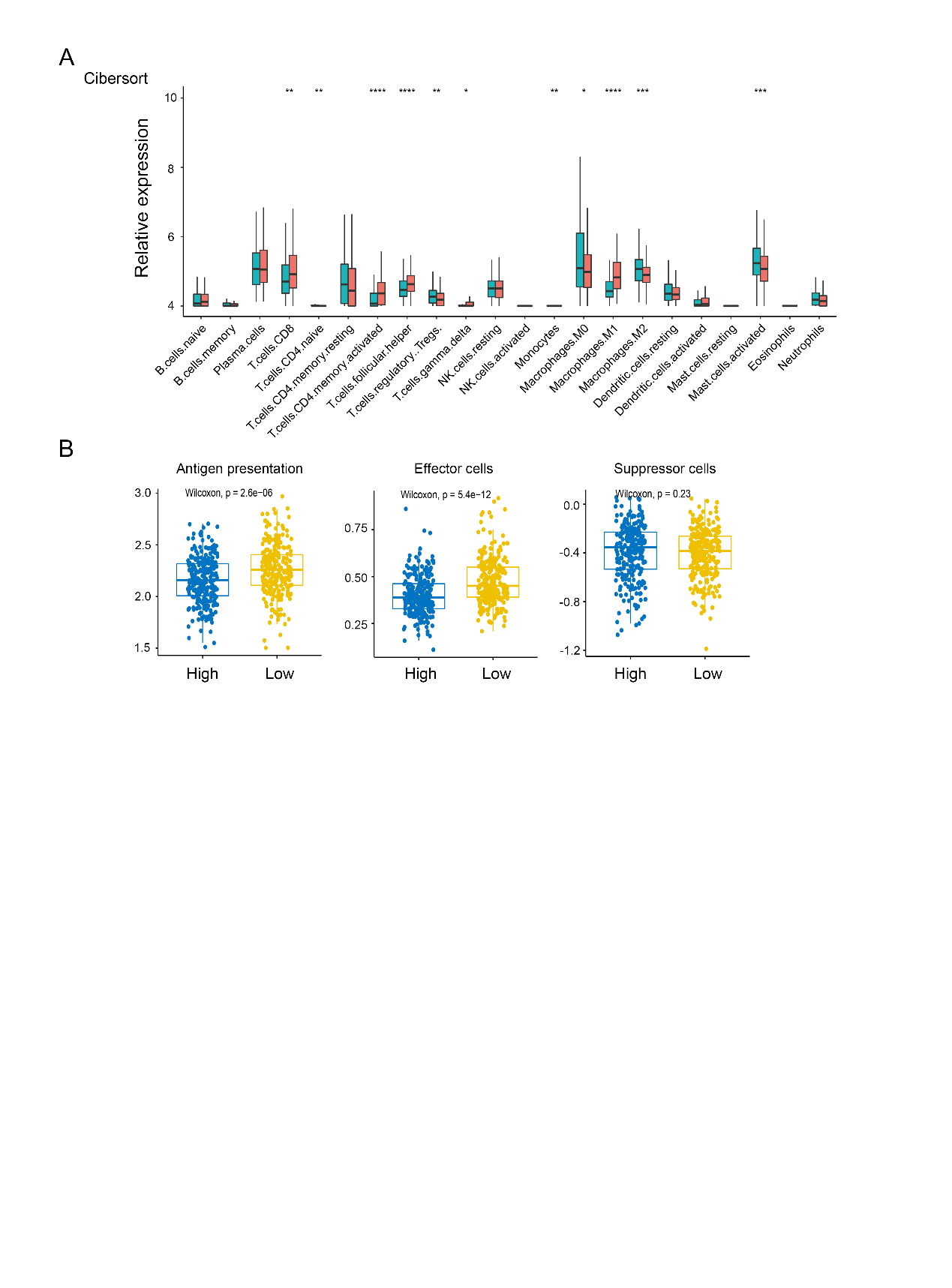


**FIGURE S3**. Evaluation of immune cells infiltration using Cibersort (A) and the antigen presentation, effector cells and suppressor cells (B) in NRG-high and NRG-low groups.


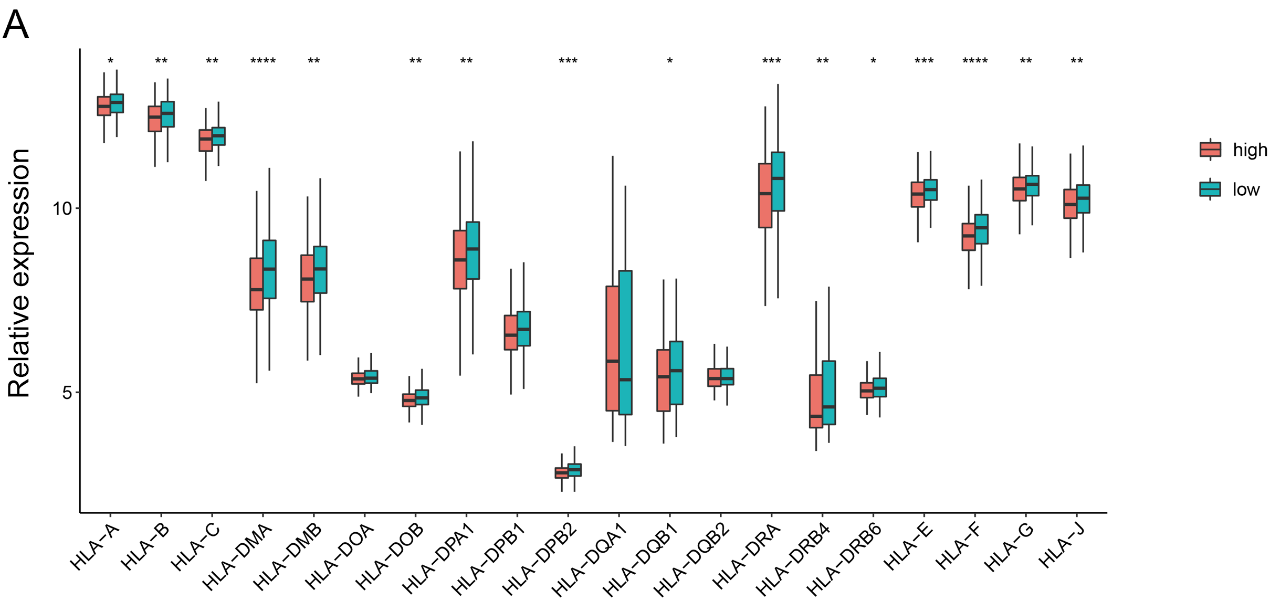


**FIGURE S4**. Gene expression of MHC between the two groups (A).


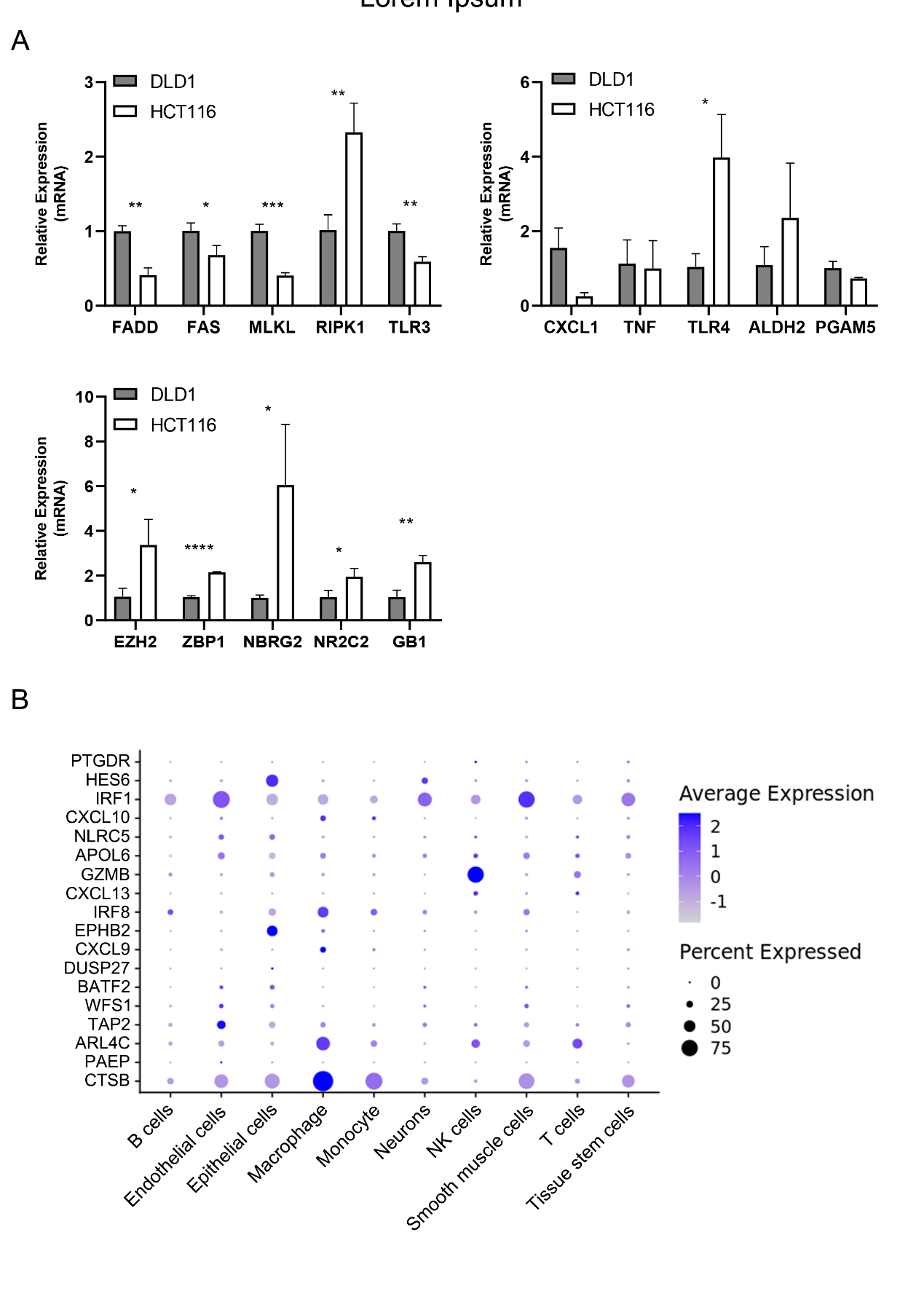


**FIGURE S5**. The mRNA expression of NRGs in different colon cancer cell lines (A) and Dot plot represented the expression of NRGs of each cell type in CRC tissues (B).

**Table. S1.** List of the GEO or TCGA databases

| Cohort | Platform | GeneChip | Samples |
| --- | --- | --- | --- |
| TCGA | RNA-seq | / | 279 |
| GSE17537 | micro-array | GPL570 | 55 |
| GSE17536 | micro-array | GPL570 | 145 |
| GSE38832 | micro-array | GPL570 | 122 |
| GSE16158 | micro-array | GPL8321 | 191 |
| GSE39582 | micro-array | GPL570 | 519 |

**Table. S2.** List of the qPCR and semi RT-PCR primer sequences

| Primer name | Primer sequence (from 5’ to 3’) |
| --- | --- |
| CTSB Forwad | GAGCTGGTCAACTATGTCAACA |
| CTSB Reverse | GCTCATGTCCACGTTGTAGAAGT |
| PAEP Forwad | GAGATCGTTCTGCACAGATGG |
| PAEP Reverse | CGTTCGCCACCGTATAGTTGAT |
| ARL4C Forwad | CCAGTCCCTGCATATCGTCAT |
| ARL4C Reverse | TTCACGAACTCGTTGAACTTGA |
| TAP2 Forwad | TGGACGCGGCTTTACTGTG |
| TAP2 Reverse | GCAGCCCTCTTAGCTTTAGCA |
| WFS1 Forwad | GTTCCCGACTCAATGCCACA |
| WFS1 Reverse | CCGCTGCGTCTCTAACACC |
| BATF2 Forwad | GCAGGGGTCTTCCTCTAAGC |
| BATF2 Reverse | GCTGCTGAGAGAGCAGGTTT |
| DUSP29 Forwad | CCAAGCTCTACATTGGCGATG |
| DUSP29 Reverse | TGTCGCGGTAGTAGTCGGG |
| CXCL9 Forwad | CCAGTAGTGAGAAAGGGTCGC |
| CXCL9 Reverse | AGGGCTTGGGGCAAATTGTT |
| EPHB2 Forwad | AGAAACGCTAATGGACTCCACT |
| EPHB2 Reverse | GTGCGGATCGTGTTCATGTT |
| IRF8 Forwad | ATGTGTGACCGGAATGGTGG |
| IRF8 Reverse | AGTCCTGGATACATGCTACTGTC |
| CXCL13 Forwad | GCTTGAGGTGTAGATGTGTCC |
| CXCL13 Reverse | CCCACGGGGCAAGATTTGAA |
| GZMB Forwad | TACCATTGAGTTGTGCGTGGG |
| GZMB Reverse | GCCATTGTTTCGTCCATAGGAGA |
| APOL6 Forwad | ACCAGGCGGAGAGAGAAAGT |
| APOL6 Reverse | TGTAGCTCCACGTCTTCACAC |
| NLRC5 Forwad | ACAGCATCCTTAGACACTCCG |
| NLRC5 Reverse | CCTTCCCCAAAAGCACGGT |
| CXCL10 Forwad | GTGGCATTCAAGGAGTACCTC |
| CXCL10 Reverse | TGATGGCCTTCGATTCTGGATT |
| IRF1 Forwad | ATGCCCATCACTCGGATGC |
| IRF1 Reverse | CCCTGCTTTGTATCGGCCTG |
| HES6 Forwad | TGACCACAGCCCAAATTGC |
| HES6 Reverse | TGACCACAGCCCAAATTGC |
| PTGDR Forwad | GGTGCTTTATCCAGATGGTCC |
| PTGDR Reverse | GTGCATCGCATAGAGGTTGC |
| GAPDH Forwad | TGCACCACCAACTGCTTAGC |
| GAPDH Reverse | GGCATGGACTGTGGTCATGAG |
